# Forces Acting on the Foot of the American Alligator (*Alligator mississippiensis*) During Pedal Anchoring

**DOI:** 10.3390/biology13121062

**Published:** 2024-12-18

**Authors:** Collin Walter, Jamie Carroll, Michael Cramberg, Jeremy J. Houser, Debra Loguda-Summers, Bruce A. Young

**Affiliations:** 1 Department of Anatomy, Kirksville College of Osteopathic Medicine, Kirksville, MO 63501, USA; sa204366@atsu.edu (C.W.); mcramberg@atsu.edu (M.C.); jhouser@atsu.edu (J.J.H.); 2 Academic Technologies, A.T. Still University, Kirksville, MO 63501, USA; jcarroll@atsu.edu; 3 A.T. Still Memorial Library, Kirksville, MO 63501, USA; dsummers@atsu.edu

**Keywords:** locomotion, biomechanics, strain, stress, trackways, footprints, reptile

## Abstract

This study was undertaken to explore the forces acting on the pes during pedal anchoring and to discern if pedal anchoring required the activation of the intrinsic pedal musculature. Replica feet equipped with strain gauges were moved over mud substrate, mimicking locomotion and pedal anchoring. Quantification of the substrate tracks demonstrated that they were similar to those made by freely moving *Alligator*, that the locomotor and pedal anchoring tracks were significantly different, and that the composition of the artificial feet significantly altered the tracks. Strain gauges revealed significantly different forces at different locations (e.g., digit vs. heel) on the pes and between locomotor and pedal anchoring motions. Collectively, the results of the present study demonstrate that the forces acting on the pes during pedal anchoring are different from those during locomotion. Furthermore, varying the composition of the feet used in this study demonstrated the importance of flexion at the metatarsal/phalangeal joints. Resistance to this flexion in living crocodylians requires active muscle contraction, meaning that pedal anchoring is an active, not passive, behavior. These results offer the first insights into the mechanics of pedal anchoring and demonstrate how technologies like 3D printing can be applied to established problems like fossil trackways.

## Simple Summary

The American alligator (*Alligator mississippiensis*) and other crocodylians brace themselves against being pulled forward by extending their hind feet (the pes) into the substrate, which is a behavior termed pedal anchoring. This study explored the mechanics of pedal anchoring by moving replica feet (which were equipped with strain gauges to detect forces) over a mud substrate to simulate locomotion and pedal anchoring. The results of this study demonstrate that (1) we could replicate the tracks made by freely moving alligators; (2) the forces acting on the pes during locomotion and pedal anchoring are significantly different; and (3) pedal anchoring required resistance to flexion at the metatarsal/phalangeal joint, indicating that it required active muscle contraction. This study demonstrates how technologies like 3D printing can be used to approach aspects of locomotion in living and extinct animals quantitatively.

## 1. Introduction

**Figure 1:**
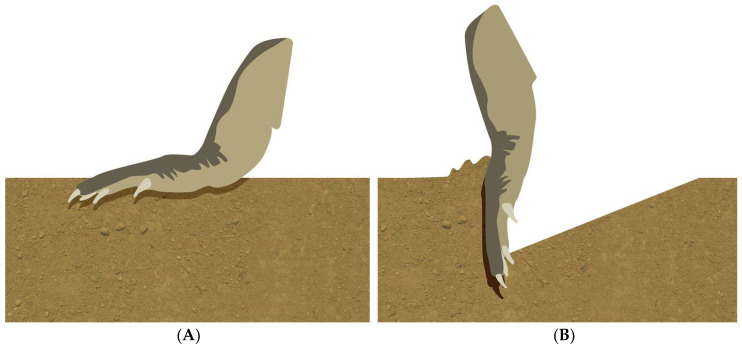
Generalized schematic of the difference between the use of the pes during walking (**A**) and pedal anchoring (**B**).

During pedal anchoring, crocodylians hold the pes (hind foot) nearly vertically, extend the digits and metatarsals into the substrate (Figure 1), and then use the dorsum of the pes as a contact point with the substrate [1]. Pedal anchoring provides the crocodylian with a tether or grapnel (the dorsum of the pes), which is used to resist the forward displacement of the body. This anchoring or attachment of the pes is fundamentally different from normal locomotor braking, which involves the plantar surface of the foot [2].

By quantifying the trackways made by *Alligator mississippiensis*, both in the wild and during laboratory trials, Walter et al. [1] demonstrated that the pedal anchoring tracks were distinct from those generated during locomotion. In addition to a suite of shape differences, the pedal anchoring tracks typically bore groove-like scratches from the dorsum of the digits and had a leading berm of substrate that was pushed up from the dorsum of the pes [1]. Pedal anchoring tracks are similar to some of the “elongate” tracks from dinosaurs that have been attributed to the animal slipping on the substrate [3,4,5].

The present study was intended not as a behavioral description of pedal anchoring but rather as an initial exploration of the interactions between the pes and the substrate during pedal anchoring. More specifically, this study examined the hypotheses that the forces acting on the pes during pedal anchoring were distinct from those acting on the pes during locomotion, that pedal anchoring was an active (not passive) behavior, particularly with regard to the motor control of the metatarsals, and that the pes/substrate interactions could be effectively investigated using artificial elements.

## 2. Materials and Methods

The earlier laboratory study of pedal anchoring [1] used a group of eight live sub-adult *Alligator mississippiensis* moving over a large mud trackway; analyses revealed that the prints made by the alligators in this mud trackway were not significantly different from tracks made by the same animals moving in the wild. This substrate was retained, maintained, and used in the same way for the present study.

Following the initial pedal anchoring study, the sub-adult *Alligator mississippiensis* were used for neurosurgical studies [6,7]. Upon completion of these (terminal) surgeries, the hind limb of a 178 cm (total length) alligator was removed at the knee. The excised hind limb was pinned in a position observed during locomotion (using video records from [1]) with the metatarsals and phalanges aligned and nearly planar and a nearly 90° flexion at the tarsal joint (ankle) and then placed in a freezer. A mold of the frozen foot was made using Alginate gel (Smaphy Inc.; Bejing, China), and then casts of the foot were made with Plaster of Paris and silicone rubber (Smooth-Sil 960; Smooth-On, Inc.; Macungie, PA, USA). The Plaster of Paris cast was scanned using Metascan (Abound Labs, Inc.; New York, NY, USA), edited with Meshmixer (Autodesk, Inc.; San Francisco, CA, USA), and then 3D printed at a 0.0050 slice height with a solid infill using an F170 printer (Stratasys) and acrylonitrile butadiene styrene (ABS) material. A (fixed) mechanical ankle and “handle” were attached to the rubber and 3D-printed models. In this way, three test feet were produced, all with identical dimensions but with a range of stiffness (3D printed > rubber > natural) that was used to represent a range of rigidity achieved by activating of the pedal musculature.

The goal of this study was to look at the forces acting between the pes and the substrate. The standard tool for achieving this is a force plate, but to quantify pedal anchoring the force plate would have to be held vertically and submerged in mud. Given the challenges of flowing substrate around a stationary force plate, we used strain gauges as an indirect measure of force. A strain gauge has a linear response, a small area, a low profile (so it should not influence the interaction of the pes and the substrate), can be readily bonded to the real and model feet, and is electrically encapsulated (so that it can work when submerged in mud). A strain gauge produces a differential voltage output depending on the change in length of the fine metal coils in the strain gauge upon the application of a force. The change in length (strain) was not the goal of this study, but since the strain gauge has a linear response, we used the differential voltage of the strain gauge as a means of measuring the force applied.

**Figure 2:**
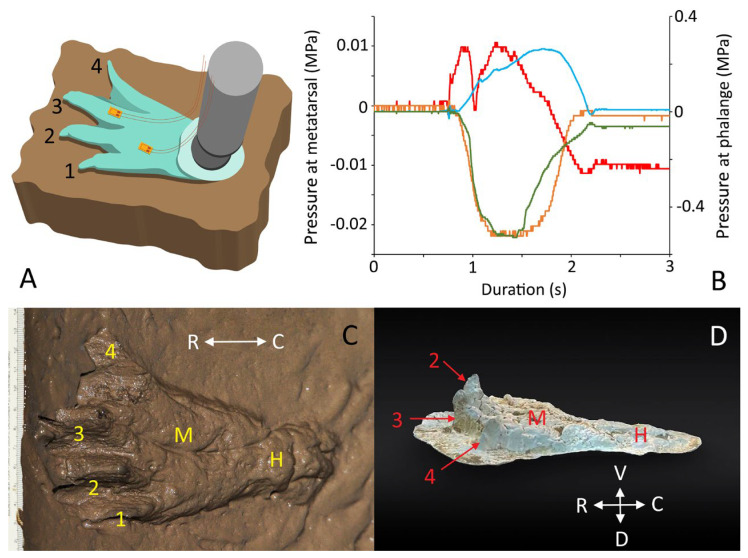
Methodology of this study. (**A**) Real and model pes (hind feet) of the American alligator (*Alligator mississippiensis*) were instrumented with strain gauges and then manually locomoted over several substrates. (**B**) The strain gauges responded to the tensile and compressive forces acting at four locations on the pes. (**C**) The footprints or trackways formed by the trial runs were cast in Plaster of Paris. (**D**) The Plaster of Paris casts were photographed (and scanned) and then imported into ImageJ, with which the features were quantified. H—heel pad; M—metatarsals; numbers indicate the digits of the alligator’s pes.

A set of four identical uniaxial strain gauges (BF350-3AA, DAOKI; Bejing, China) was superglued on the same location of each test foot: the dorsum of the shaft of the proximal phalange of the 3rd digit, the plantar surface of the shaft of the proximal phalange of the 3rd digit, the dorsum of the pes over the 2nd metatarsal, and the plantar surface of the pes over the 2nd metatarsal (Figure 2A). The strain gauges were connected to identical Wheatstone bridges and then to P122 strain gauge amplifiers (GRASS; West Warwick, RI, USA). The signals from the four-strain gauge amplifiers (Figure 2B) were recorded simultaneously (at 4 kHz) using the MiDAS data acquisition system (XCitex, Inc; Woburn, MA, USA).

Each test foot was moved over the substrate manually by the same investigator 20 times, including 10 times in a fashion intended to replicate a heel-to-toe locomotor step and 10 times in a fashion intended to replicate pedal anchoring. The movements of the test foot relative to the substrate were based on video recordings of the live alligators moving over the mud trackway [1]. The 60 tracks produced (Figure 2C) in this way (3 test feet, 10 steps each, 10 pedal anchors each) were cast with Plaster of Paris (Figure 2D) and then photographed and/or 3D scanned (Metascan). The digital images were imported into ImageJ (NIH), and the depth of the cast was quantified at three anatomical “levels”: the distal tip of the 2nd digit, the center of the heel pad, and the mid-point of the “arch” (corresponding to the metatarsals). 

**Figure 3:**
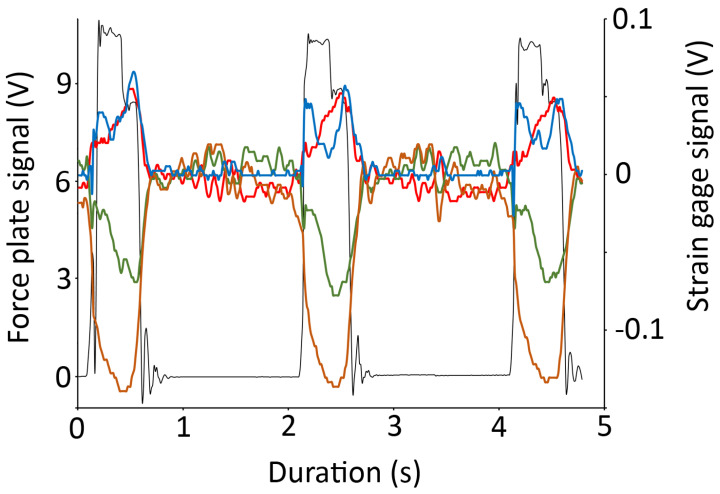
Raw data traces from a natural foot “walking” over the force plate. Simultaneously recorded voltage output from the force plate (black trace) and the four strain gauges (dorsal surface of the proximal 3rd phalange—blue; plantar surface of the proximal 3rd phalange—green; dorsal surface of the 2nd metatarsal—red; and plantar surface of the 2nd metatarsal—orange) demonstrate that even though the foot was “walked” manually, it resulted in fairly consistent signals. Note that the force plate operated over a different voltage scale (left y-axis) than the strain gauges (right y-axis).

A simple force plate was constructed with a 20 × 20 cm plate of aircraft aluminum and an FT10 force transducer (GRASS). Calibration of the force plate using weights of known mass confirmed that it had a linear response. Each test foot was moved over the force plate in a step-like fashion 10 times. Each time the test foot contacted the force plate, we simultaneously recorded two sets of data: voltage out from the force plate and voltage output from the strain gauges (Figure 3). Since the force plate had been calibrated, the quantitative relationship between the two simultaneous voltage signals (force plate and strain gauge) was used to calibrate the strain gauge signal to the applied force. To be as robust as possible, the strain gauge records were exported to EXCEL, where the peak, area under the curve, and root mean square (RMS) were determined for each record (in volts); the area under the curve and RMS values were also standardized to a one-second curve duration. Since the strain gauge attachment sites on the test feet had different cross-sectional areas (1 cm^2^ for the phalange and 4.5 cm^2^ for the metatarsal), the calibrated output from the strain gauges (the force) was divided by the cross-sectional area (thus yielding a pressure).

Each combination of the 3 feet (natural, rubber, and 3D printed), 3 movements (force plate, walking, and pedal anchoring), and 4 strain gauge metrics (area under curve, area/1s, RMS, RMS/1s) was repeated 10 times. The data were screened by taking the mean and standard deviation (s.d.) for the 10 replications. Data points were excluded from analysis only if (1) they exceeded ±1.5 s.d. from the mean, and (2) the pressures on opposite surfaces (e.g., dorsal and plantar phalange) were not ±1.5 s.d. away from the mean value at that recording site. Three rounds of two-tailed MANOVA were conducted, as follows: (1) each recording site (e.g., dorsal phalange) was tested across the three feet and three movements; (2) each foot was tested across the four recording sites and three movements; and (3) each movement was tested across the four recording sites and three feet. Each round of MANOVA was repeated four times, once for each metric of pressure (e.g., peak, RMS). Differences, whether the rows and columns (e.g., between feet) or the subsequent Tukey’s HSD comparisons, were all made with Bonferroni-adjusted *p* values. A difference (e.g., dorsal vs. plantar phalangeal pressure on the rubber foot during walking on the substrate) was only deemed significant if the calculated *p* value was less than the Bonferroni-adjusted threshold on at least three of the four metrics of pressure. The RMS/1s pressure values for pedal anchoring and walking over the mud substrate were subsequently Z-transformed and used as input for a principal component analysis (using Q version 5.4.1, Research Software). 

A complete tabulation of statistical tests and results is given in the Supplementary Materials.

## 3. Results

**Figure 4:**
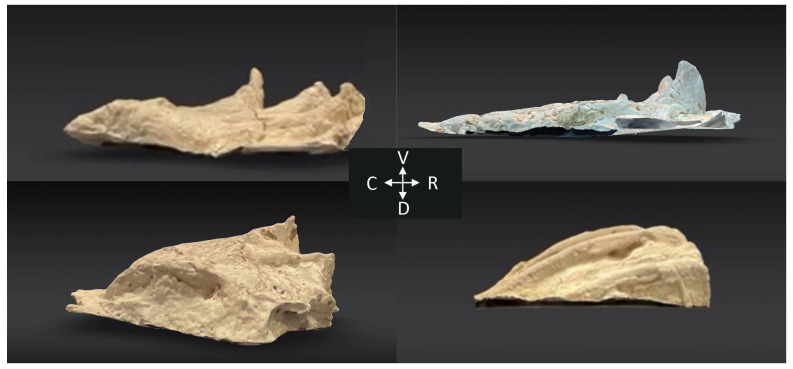
Three-dimensional scans of representative casts. The two upper casts were made during walking, the two lower casts were produced during pedal anchoring. The casts on the left are from freely moving live *Alligator mississippiensis*; the casts on the right were made by manually moving model pes.

**Figure 5:**
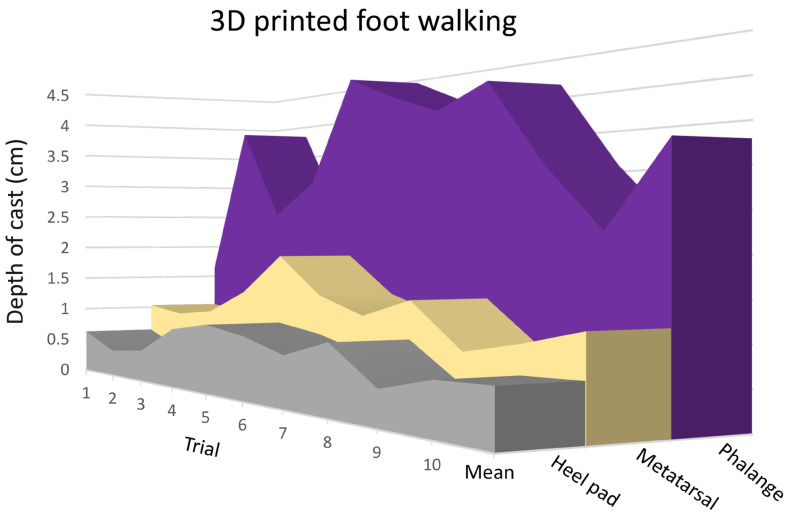
Data from the 10 walking trials involving manually moving a 3D printed pes. Note that although these movements were made manually, there is consistency in the data both in the pattern (phalanges always have the deepest penetration, and the heel pad always has the shallowest) and across the trails (increases in heel pad depth are mirrored by increases in metatarsal and phalangeal depth). The mean of the ten trials is presented on the far right.

The tracks and associated casts produced during this study were similar in appearance (Figure 4) to those made by the live animals [1]. The mean values of the three metrics taken from each cast (Table 1) were within the range of what was reported from the recent study of live alligators [1]. The tracks analyzed for the present study were made manually, but care was taken to keep the motion (and thus the resulting track) as consistent as possible (Figure 5).

MANOVA demonstrated that there was a significant (F = 46.38, df = 8, *p* = 1.11 × 10^−16^) difference among the dimensions of the casts produced during the simulated walks. A post hoc Tukey’s analysis revealed that the significant differences involved greater phalangeal depth than metatarsal or heel pad depth from each trial foot. The same measure (e.g., heel pad depth) was not significantly different among the three trial feet. 

A MANOVA demonstrated that there was a significant (F = 77.39, df = 8, *p* = 1.11 × 10^−16^) difference among the dimensions of the casts produced during the simulated pedal anchoring. A post hoc Tukey’s analysis revealed that the significant differences involved both the metatarsal and phalangeal depth measurements from each trial foot. The heel pad depth and metatarsal depths were not significantly different between the three trial feet; however, there were significant differences among the phalangeal depths.

**Figure 6:**
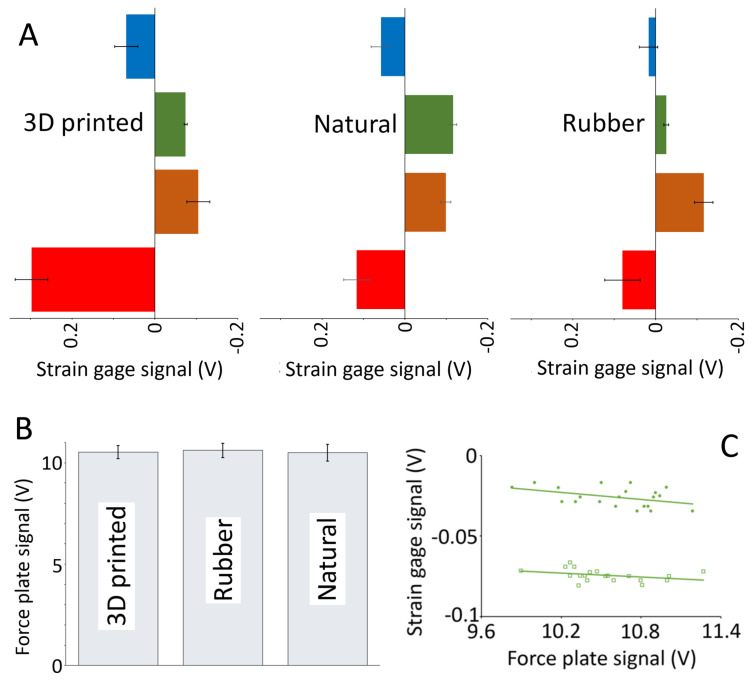
Relative variation in the strain gauge data. Each pes was instrumented with four strain gauges positioned on the dorsal surface of the proximal 3rd phalange (blue), the plantar surface of the proximal 3rd phalange (green), the dorsal surface of the 2nd metatarsal (red), and the plantar surface of the 2nd metatarsal (orange). (**A**) Each pes was then manually moved (in this case, in a walking motion over a force plate) for ten trials; the bar width is the mean, while the error bars display ±1 standard deviation. (**B**) The corresponding force plate signals; note the difference in the y-axis between the strain gauges and the force plate. There is relative consistency in the data, in both the forces applied to the force plate when manually moving the pes, and in the resulting pattern of four discrete strain gauge voltages (one from each recording site) on each pes. Some of the variation denoted by the error bars reflects the linear relationship that was found between each strain gauge signal and the force plate signal. (**C**) The relationship between these two for the plantar surface of the proximal 3rd phalange on the rubber pes (solid circles) and the 3D printed pes (open squares).

**Figure 7:**
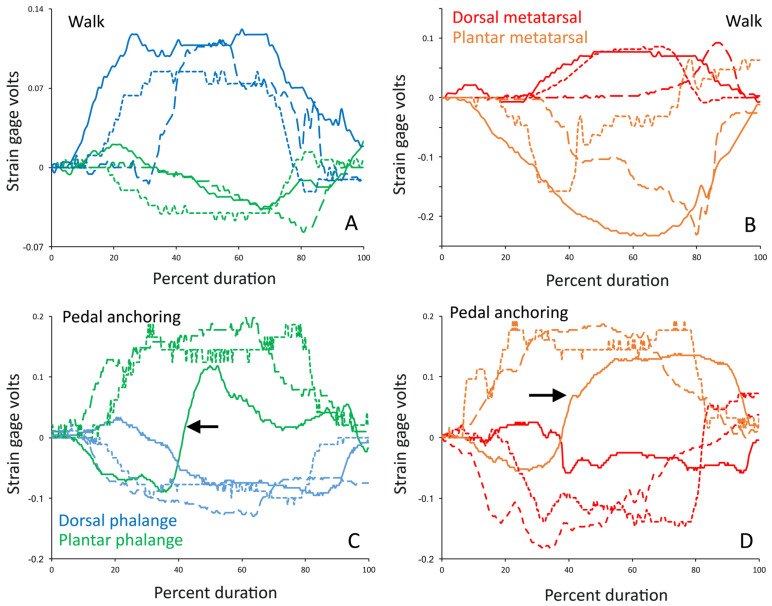
General pattern of the strain gauge response during the manual movement of the pes of *Alligator mississippiensis*. These are unfiltered traces. (**A**,**B**) are data traces from walking trials; (**C**,**D**) are data traces from pedal anchoring trials. (**A**,**C**) are data from the phalange; (**B**,**D**) are data from the metatarsal. Solid lines were recorded from the natural pes, long dashed lines were recorded from the rubber model, and short dashed lines were recorded from the 3D printed model. Note the general agreement in pattern between the three models. In both the phalanges and the metatarsals the values switch from positive to negative, indicating a switch from tension to compression, when switching from walking to pedal anchoring. During pedal anchoring, the signals from the plantar surface of the natural foot are more complex, reflecting both tension and compression (black arrows) as the toes deflect and drag along the substrate.

Like the trackways (Figure 5), the manual movements of the trial feet produced consistent patterns and levels of pressure (Figure 3 and Figure 6). There was a consistent pattern observed in the pressures. During walking, the dorsal surface of the phalanges experienced compression of a magnitude that was approximately 1.5× the level of tension that was recorded from the plantar surface of the phalange (Figure 7A). The dorsal surface of the metatarsals experienced compression, while the plantar surface of the metatarsals experienced tensions that were 3–4× the magnitude (Figure 7B). During pedal anchoring, the dorsal surface of the phalanges experienced tension, while the ventral surface of the phalanges experienced compressions that were roughly 1.5× the magnitude of the tensions. The ventral phalanges of the natural foot were unique in that they were initially exposed to tensions and then switched to compressions, with both of nearly equal magnitude (Figure 7C). The dorsal metatarsals experienced tension during pedal anchoring, while the plantar surface of the metatarsals experienced compression of similar magnitude (Figure 7D). The ventral metatarsals of the natural foot were unique in that they were initially exposed to tension and then switched to compression, with the compression being roughly twice the magnitude (Figure 7D).

**Figure 8:**
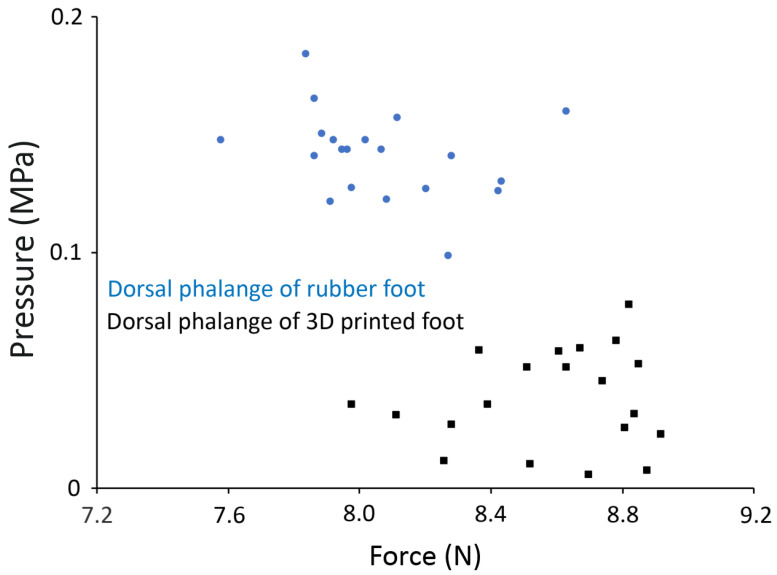
Relative consistency and differentiation of the pressure values. The pressure determined from the dorsal surface of the 3rd phalange during ten walking trials of the rubber pes (blue circles) and the 3D printed foot (black squares). These two model feet are the same size, and the strain gauges are positioned the same way, yet movement over the same force plate yields two discrete (and significantly different) pressure levels. Note that during the trials involving the 3D printed pes, slightly higher forces were applied (x-axis), yet the pressures recorded at the phalange were slightly lower (y-axis).

The general patterns illustrated in the raw voltage traces in Figure 7 do not take into account the differential areas of the phalanges (1.0 cm^2^) relative to the metatarsals (4.5 cm^2^) nor the specific calibration of each strain gauge. These factors were taken into account, the different metrics for quantification were applied, and the resulting mean values for the 10 trials are given in Table 2. As evident in Table 2, there were differences in both the direction (tensile versus compressive) and magnitudes of the pressures recorded. Many of these differences were evident even when pressures recorded from the same location were compared during the same type of movement (Figure 8). 

MANOVAs with Bonferroni-adjusted significance levels were used to compare the pressures (with restrictions detailed above). Overall, significant differences were found in 80% of the analyses (Table 3). This high level of significant differences reflected the fact that all of the analyses between different recording locations and all of the analyses between different movements were significant. The frequency of significance in the post hoc (Bonferroni-adjusted) Tukey’s analyses varied widely (Table 3) but were generally lowest for the comparisons between the three trial feet.

As an additional means of examining the differences between the three feet models, the RMS/s pressure values for the dorsal phalange and dorsal metatarsal were compared; in effect, this meant looking for changes in pressure level over the metatarsal/phalangeal joint (Table 4). This revealed differences not only in the magnitude but also in the direction of changing pressure at this joint. The calculated differential values were used to form a 3 × 3 MANOVA. The results revealed that the natural foot had significantly different pressure patterns across the joint than either the rubber or 3D-printed feet (which were not significantly different) and that pedal anchoring produced a pattern of pressure across the joint that was significantly different from what was found during walking on either the force plate or mud substrate (which were not significantly different).

**Figure 9:**
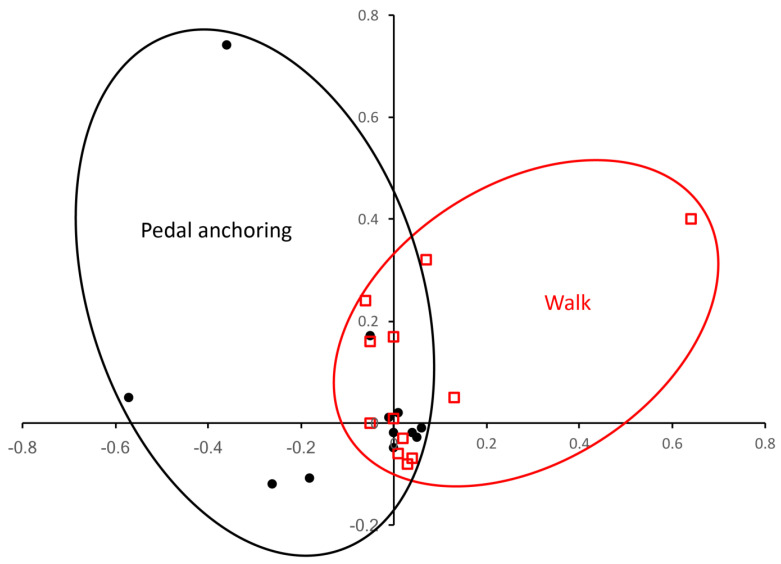
Results of the principal component analysis (PCA) of the pressure values are shown; the first component (horizontal axis) and third component (vertical axis) account for approximately 66% of the variation. There is a population of walking pressures (red) and a population of pedal anchoring pressures (black) that overlap in a zone of minimal pressure differentiation.

**Figure 10:**
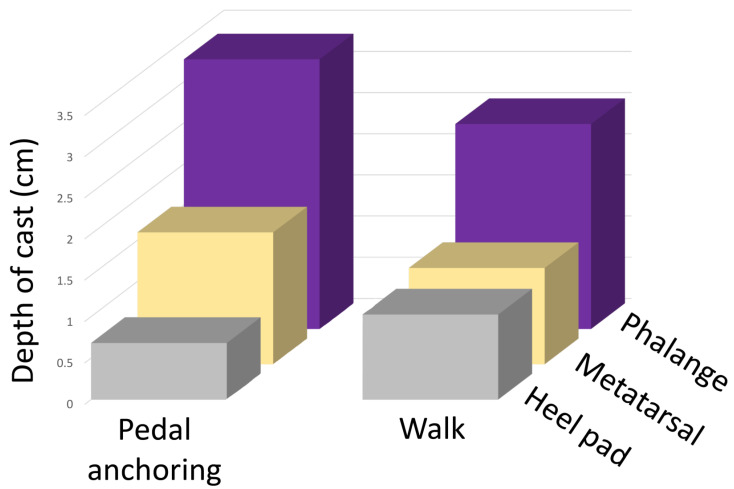
Ten casts taken from the walking trials with the highest PCA eigenvector scores were compared to ten casts taken from the pedal anchoring trials with the lowest PCA eigenvector scores. The mean values for the casts, all of which were significantly different, are depicted by the height of the columns.

A principal component analysis (PCA) of the Z-transformed pressures for the three trial feet walking on mud and pedal anchoring in mud produced two overlapping groups of characters (Figure 9), explaining some 62% of the variation in pressure values. The Eigenvectors (component scores) for this analysis (see the Supplementary Materials) were used to identify 10 trials with maximal “Walk” PCA pressures and 10 trials with maximal “Pedal anchoring” PCA pressures. The casts from those 20 trials were then compared. A MANOVA found a significant (F = 122.65, *p* < 0.0001) difference between the walk and pedal anchoring casts, and Tukey’s post hoc analysis found significant differences between the depth of the heel pad, the metatarsal depth, and the depth of the phalanges (Figure 10).

**Figure 11:**
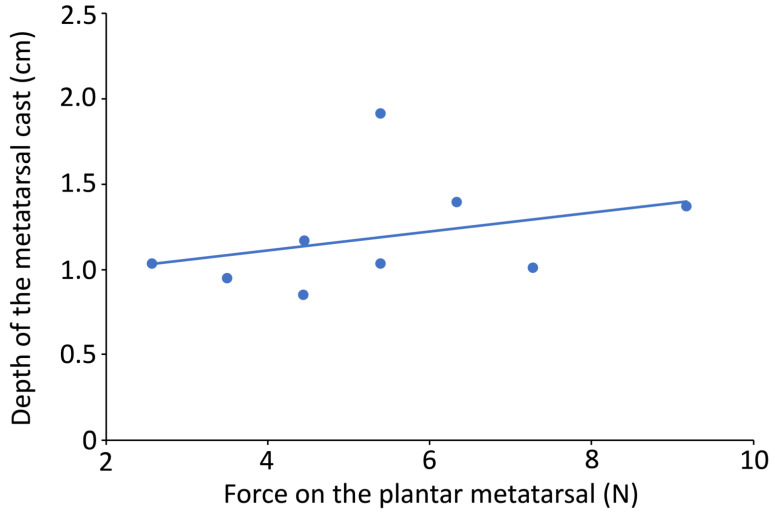
Plot of the depth of the metatarsal region of the cast (y-axis) from ten tracks made by manually “walking” a 3D printed pes over mud. The force applied at the plantar region (x-axis) of the pes has a clear relationship to the substrate deformation (as measured by cast depth on the y-axis).

Using the determined relationship between strain gauge signal voltage and applied force (recorded by the force plate), it is possible to look at the relationship between applied force and cast dimensions (Figure 11). The relationship between applied force and cast dimensions will be not only model-specific but also very substrate-specific. 

## 4. Discussion 

The core methodology of this study was using detached alligator feet and models of alligator feet as substrate indenters. This is not a novel approach (for a review see [8]); Ref [9] used detached emu feet, while model feet have been used in other studies [10,11]. When coupled with video recordings (as in this study), detached and/or model feet can be moved over the surface in a “natural” way that produces tracks closely resembling those found in nature (Figure 2). The metrics of the artificial tracks made in the present study all fell within the range of those recorded from *Alligator mississippiensis* tracks collected in the wild [1].

Walter et al. [1] described pedal anchoring, a behavior performed by *Alligator mississippiensis* in which the pes (hind foot) is extended into the substrate and contact between the dorsum of the foot and the substrate forms an anchoring point for the animal. During the lift-off phase of walking, the phalanges of the *Alligator* pes typically flex; during pedal anchoring, the dorsum of the pes is dragged along the substrate (producing distinctive digital scratches) and then the pes flexes at the ankle to increase the contact area perpendicular to the substrate [1]. These different movements result in significantly different tracks both in the wild and in controlled laboratory trials [1]. This analysis used the same substrate as the earlier study, used the detached pes of one of the alligators from the earlier study [1], and like the earlier study, found significant differences in the tracks produced during walking and pedal anchoring (Figure 9). As was found in the earlier study on natural tracks, the largest difference was in the range (and direction) of the phalanges (Figure 4). “Rolling” the artificial pes over the planted phalanges during lift-off was enough to drive the digits (caudally) into the substrate, replicating the digital flexion that occurs during walking; this could be performed similarly in the three different model feet, so no significant differences were found in the dimensions of the walking casts made by the different models. During pedal anchoring, the pes is extended into and partially rotated (rostrally) through the substrate (to maximize perpendicular contact area); this movement was easiest to replicate using the stiff (3D printed) pes and most difficult with the more pliant detached pes. Unlike the casts made during simulated walking, the casts made during simulated pedal anchoring had significant differences among the three model feet and significant differences in phalangeal depth. Previous work on emu locomotion and the resulting tracks [12] has similarly shown that changes in the gait or acceleration of the animal can result in distinctive changes in the resulting track.

By definition, when a terrestrial animal changes gait, there is a difference in the forces acting on the pes. This difference has been shown using force plates and gait transitions in a variety of animals, including chickens [13], kangaroo rats [14], and horses [15]. Other researchers have shown this force difference by outfitting humans [16] and horses [17,18] with sensory-equipped shoes. The differential forces associated with gait transitions have also been shown using strain gauges in horses [15], dogs [19], and sheep [20], among other taxa. 

In the present study, although the model feet were moved manually, the data within each gait were consistent (Figure 5). A conservative protocol was employed to determine significant differences in the data set; nevertheless, the walking and pedal anchoring data from each of the three trial feet were found to be significantly different. Pedal anchoring is a behavior that places different forces on the pes than occurs during walking. When PCA was used to differentiate the walking and pedal anchoring trials (Figure 9), the trials with the strongest discriminant scores yielded casts that were significantly different on all quantified features. Therefore, the significantly different pressures acting on the model feet during walking and pedal anchoring reflected differential forces from the substrate that were consistent enough to yield significantly different tracks (casts). 

The three model feet used in this study were identical in size and shape. Furthermore, the two replica feet had been reproduced with such fidelity that they exhibited the same scalation pattern as the detached foot. The key difference between them was in their relative flexibility. The 3D-printed foot was rigid, and the cast rubber foot was more flexible, particularly in the digits (where the cross-sectional area was lower). The detached foot was the most flexible, again, particularly at the phalangeal joints, due to the absence of intrinsic skeletal muscle control. 

The locomotor steps started with heel planting, and then the foot was effectively “rolled” over the digits; accordingly, the substrate contact was always on the ventral surface of the foot. During walking, the digits exhibited little flexion; this was due to the composition of the 3D-printed and cast rubber feet and the phalangeal joints of the detached foot, which afford a greater range of ventroflexion compared to dorsoflexion. During pedal anchoring, the dorsal surface of the pes was typically dragged over the substrate (producing distinctive digital grooves [1]), and then the pes was rotated dorsally about the ankle and the leg extended to drive the dorsal surface of the pes against the substrate (so much that berms of the substrate formed on the dorsal surface of the pes [1]). This behavior was easy to replicate with the stiff 3D-printed foot and the slightly more flexible cast rubber foot. The flexibility of the digits of the detached foot meant that on the ventral palmar surface of the foot, there was a marked transition when the foot was rotated; the rotation through the substrate caused ventroflexion of the digits and a corresponding switch from tension to compression (Figure 6). This unique transition from tension to compression on the detached foot is the main reason that greater levels of difference were observed between the pedal anchoring trials compared to the walking trials (Table 2 and Table 3). 

**Figure 12:**
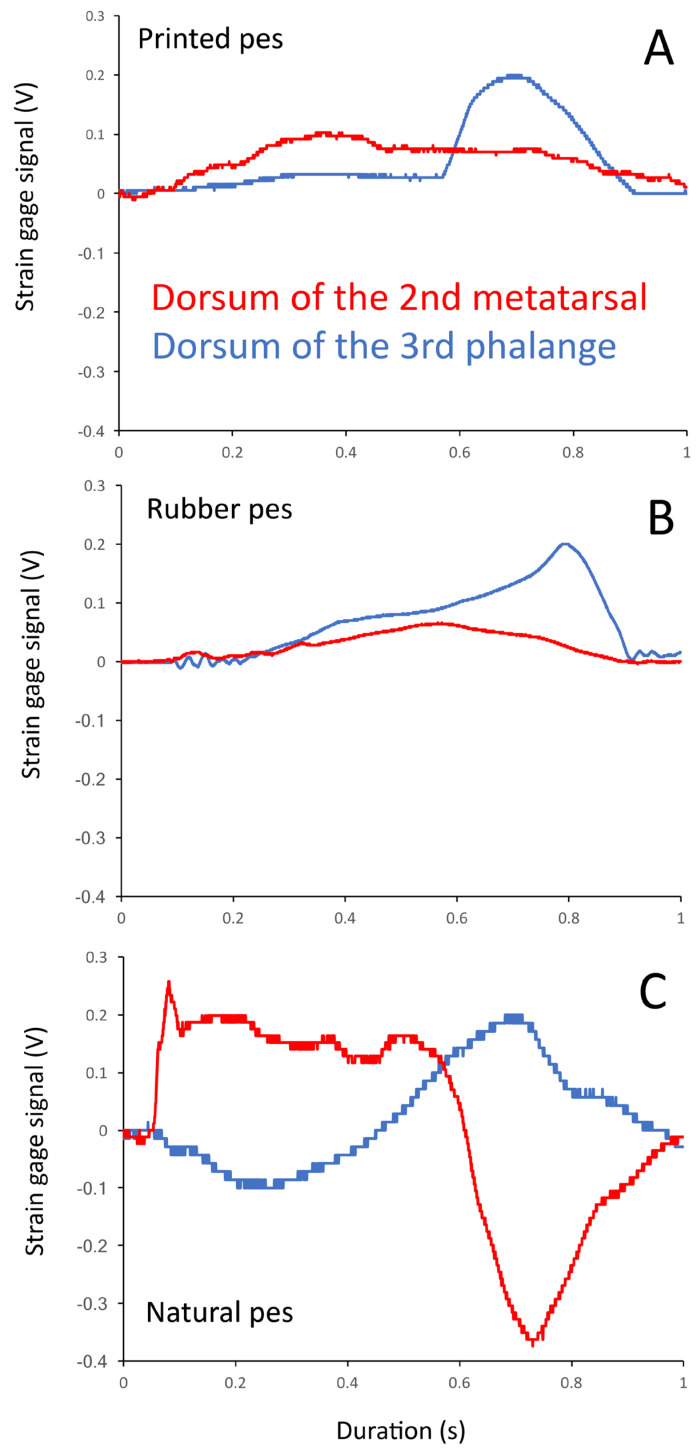
Strain gauge recordings from the 3D-printed pes (**A**), cast rubber pes (**B**), and natural pes (**C**) moving over the force plate. The traces were mathematically adjusted to have a common duration of 1 sec and to have the peak voltage from the phalangeal strain gauge = 0.2 volts. Note the relative similarity in the traces between the 3D-printed and cast rubber traces (**A,B**) and the greater variation/complexity recorded when the same movement is made with the natural foot (**C**).

Turner and Gatesy [21] demonstrated that during locomotion, alligators could modulate their intertarsal mechanics to maintain a plantigrade contact; pedal anchoring was not included in their study. The present study was designed, in part, to look at variations in the flexibility at the metatarsal–proximal phalangeal joint. By comparing the magnitudes of pressures on either side of this joint (Table 4), it can be seen that the greater flexibility in the cast rubber pes created a greater loss of pressure across the joint compared to the 3D-printed foot (the stiffness of which led to a rather constant pressure across the “joint”). The pressure pattern in the detached foot is more complicated. The mobility documented earlier [21] was present in the intertarsals, and there was mobility at the tarsal/metatarsal joints, as well as the metatarsal/proximal phalangeal joints. This mobility within the natural foot consistently made the pressure patterns recorded from the natural pes more complex than those recorded from the two model feet (Figure 12). The same basic pattern of pressure was found in the detached foot (e.g., pressure was greater on the force plate than on the natural substrate, direction of pressure changed between walking and pedal anchoring), but the patterns of the pressure, particularly on the dorsal metatarsal (Figure 12), made the pressure transfer in the detached foot markedly different from that of the two model feet (Table 4). The relationship among mobility, joint range, and force levels has been studied most extensively in human feet [22], but other researchers have taken a more comparative approach, showing the diversity of the range of motion/strain patterns associated with various pedal joint morphologies in living [23] and extinct [24] vertebrates.

The results of the strain gauge analysis, while clearly demonstrating that different pressures act on the pes during walking and pedal anchoring and that the joint mobility of the natural foot alters the pressure patterns, also suggest a fascinating avenue for future research. The rubber foot cast in this study had a consistent flexibility that could replicate aspects of the trackways but had pressure patterns significantly different from the detached foot. Similar results were obtained with the 3D-printed pes. However, the 3D-printed model could be readily modified. Using multi-media 3D printing, a pes (or other biological tissue) could be modeled as a combination of different biomechanical properties and then printed with a combination of media offering a range of strength (e.g., polycarbonate) and flexibility (e.g., thermoplastic polyurethane) so that while the size and shape of the object did not vary, the number, location, and flexibility of the intrinsic joints could [25,26]. 

The primary goal of this study was to test the hypothesis that different forces acted on the alligator pes during walking and pedal anchoring. The analysis presented herein produced significant support for the hypothesis. It bears repeating that while strain gauges were used in this study, it was not designed to examine strain in the crocodylian foot or the biomechanics of locomotion in alligators. Pedal anchoring is unusual in that it involves the active use of the dorsum of the pes in a way that results in different levels of patterns of forces acting on the pes than are normally encountered during walking. The dorsum of the manus and pes of terrestrial vertebrates are relatively understudied and may provide insight into fossil trackways and functional specializations.

## 5. Conclusions

In this study, model feet were used to study the pedal anchoring behavior of *Alligator mississippiensis*. During pedal anchoring, the pes is inserted into the substrate, and the dorsum of the pes is used as a contact point to brace the animal; previous work has shown that this behavior results in unique tracks in the substrate. The present study was designed to examine if pedal anchoring also resulted in unique forces acting on the pes. The pedal anchoring behavior was replicated using model feet (with differing flexibilities) equipped with strain gauges. Four major findings from this study are as follows: (1) trackways produced by manually moving the model feet were similar to those produced by living alligators during normal movement; (2) significantly different pressure patterns were recorded during “walking” compared to during “pedal anchoring”; (3) the pressures acting on the model feet were significantly related to quantitative features of the trackways made during “walking” and “pedal anchoring”; and (4) significant differences in pressures were found among the different model feet, which result from the different flexibilities of the model feet, particularly at the metatarsal–proximal phalange joint. 

## Figures and Tables

**Table 1:** The depth of the track and the resulting height of the cast were measured at the heel pad (gray), metatarsal region (yellow), and phalange tip (purple). The mean values for the three trial models (3D printed, rubber, and natural) are given; note the overlap between these means and the range of values recorded when the same-size specimens of *Alligator mississippiensis* were analyzed in the wild and on mud trackways in the lab. All values are in cm.

	Heel Pad	Metatarsal	Phalange
**Walking tracks**			
Live			
wild	0.18–1.08	0.24–1.68	1.36–4.07
lab	0.17–2.21	0.36–2.74	1.87–5.95
Models			
printed	0.69	1.21	3.3
rubber	1.08	1.4	3.09
natural	1.22	1.59	2.92
**Pedal anchoring tracks**			
Live			
lab	0.68–5.12	1.0–5.32	1.58–7.10
Models			
printed	0.75	1.4	3.76
rubber	0.67	1.51	3.75
natural	0.69	1.49	2.72

**Table 2:** Summary of the strain gauge data. Pressures were recorded in rubber model pes (green), 3D-printed model pes (tan), and natural pes (pink). The three models were manually walked over a force plate, manually walked over a mud substrate (“walk” in this table), and used to perform pedal anchoring in the mud substrate. The pressures (given as MPa) from the four recording sites were calculated using four different methods (left columns). The “Force transducer” rows are the values from the force plate and are given in Newtons; duration values are given in seconds. Mean values for the ten trials are presented.

			Force Plate			Walk		Pedal Anchoring		
		Rubber	3D Print	Natural	Rubber	3D Print	Natural	Rubber	3D Print	Natural
**Dorsal phalange**	Area	0.063	0.011	0.021	0.066	0.018	0.054	−1.200	−0.052	−0.188
	Area/s	0.105	0.023	0.034	0.056	0.022	0.025	−0.335	−0.016	−0.048
	RMS	0.141	0.039	0.043	0.086	0.028	0.030	−0.370	−0.020	−0.057
	RMS/s	0.225	0.076	0.069	0.051	0.030	0.036	−0.109	−0.007	−0.025
**Ventral phalange**	Area	−0.024	−0.019	−0.030	−0.124	−0.016	−0.070	0.277	0.074	0.268
	Area/s	−0.038	−0.039	−0.048	−0.060	−0.019	−0.053	0.083	0.017	0.072
	RMS	−0.045	−0.060	−0.055	−0.131	−0.016	−0.070	0.096	0.020	0.089
	RMS/s	−0.071	−0.122	−0.090	−0.105	−0.023	−0.059	0.029	0.006	0.019
**Dorsal metatarsal**	Area	0.018	0.007	0.047	0.033	0.011	0.077	−0.105	−0.030	−0.268
	Area/s	0.030	0.014	0.074	0.022	0.013	0.051	−0.024	−0.009	−0.067
	RMS	0.037	0.016	0.094	0.031	0.011	0.063	−0.027	−0.011	−0.079
	RMS/s	0.058	0.032	0.153	0.012	0.013	0.037	−0.006	−0.003	−0.016
**Ventral metatarsal**	Area	−0.026	−0.010	−0.016	−0.196	−0.207	−0.111	0.131	0.050	0.182
	Area/s	−0.041	−0.020	−0.026	−0.084	−0.252	−0.081	0.036	0.015	0.045
	RMS	−0.044	−0.021	−0.029	−0.089	−0.315	−0.105	0.041	0.019	0.055
	RMS/s	−0.072	−0.039	−0.047	−0.054	−0.270	−0.070	0.011	0.006	0.009
**Force transducer**	Area	4.812	3.205	2.087						
	Area/s	7.529	6.407	3.368						
	RMS	8.065	8.543	4.040						
	RMS/s	12.820	17.390	6.542						
**Duration**		0.635	0.495	0.617	1.889	0.888	1.452	3.596	3.410	3.737

**Table 3:** Summary of the MANOVA tests. Each comparison was run four times, each time using a different method for quantifying pressure. A significant difference was recorded only if at least three of the four tests revealed a level of significant difference (relative to a Bonferroni-adjust threshold); this value is noted in bold. In those tests in which significant differences were detected, a post hoc Tukey’s analysis was performed to determine which of the multiple comparisons were different (the frequency of significant differences among the comparisons is listed for each group of tests).

Differences Among the Three Feet (3 Out of 7 Tests, 43%)	
Rubber—3D printed	(3 out of 7, 43%)
Rubber—Natural	(2 out of 7, 29%)
3D printed—Natural	(2 out of 7, 29%)
**Differences among movements (7 out of 7 tests, 100%)**	
Substrate walk—Force plate walk	(3 out of 7, 43%)
Substrate walk—Pedal anchoring	(5 out of 7, 71%)
Force plate walk—Pedal anchoring	(5 out of 7, 71%)
**Differences among strain locations (6 out of 6 tests, 100%)**	
Dorsal phalange—Plantar phalange	(3 out of 6, 50%)
Dorsal phalange—Dorsal metatarsal	(3 out of 6, 50%)
Dorsal phalange—Plantar metatarsal	(4 out of 6, 67%)
Plantar phalange—Dorsal metatarsal	(5 out of 6, 83%)
Plantar phalange—Plantar metatarsal	(2 out of 6, 33%)
Dorsal metatarsal—Plantar metatarsal	(6 out of 6, 100%)

**Table 4:** Pressure transfer between the phalangeal and metatarsal levels of the pes. Pressures were recorded in rubber model pes (green), 3D-printed model pes (tan), and natural pes (pink). For each combination of model pes (vertical) and motion (horizontal), the mean pressure for the dorsal surface of the phalange (top) and metatarsal (middle) is given, as is the percent difference in pressure between the dorsal phalange and dorsal metatarsal (bottom).

		Natural	Rubber	3D Print
**Dorsal phalange**	force plate	0.069	0.225	0.076
	walk	0.036	0.051	0.030
	pedal anchor	−0.025	−0.019	−0.007
**Dorsal metatarsal**	force plate	0.153	0.058	0.032
	walk	0.037	0.012	0.013
	pedal anchor	−0.016	−0.006	−0.003
**Strain transfer**	force plate	221.739	25.778	42.105
	walk	102.778	23.529	43.333
	pedal anchor	64.000	31.579	42.857

## Data Availability

This study generated two interconnected data sets. The first data set is a large number of Plaster of Paris casts of the tracks (footprints) made during this study. Although we have digital scans (see the Supplementary Materials) and photographs of each cast, these may not show every feature. The size, number, and fragility of these casts preclude shipping them to a public archive. They are currently all indexed and curated. The cast themselves or specific scans/photos are available upon request to the corresponding author. The second data set consists of the data records from the strain gauges and/or force plate (see the Supplementary Materials). These were recorded using proprietary software (MiDas) but can be exported to EXCEL. These EXCEL files are generally quite large (due to the high sampling rate). Any portion of the digital data record is available upon request to the corresponding author.

## Supplementary Materials

The following supporting information can be downloaded at https://www.mdpi.com/article/10.3390/biology13121062/s1: File 1: Guide to the Supplementary Materials; File 2: Metascan_20240917-0854, File 3: Statistical Summary; File 4: Supplementary Materials EXCEL data; File 5: Supplementary Materials screenshots.
