# Supplementary material for: Forces Acting on the Foot of the American Alligator (Alligator mississippiensis) During Pedal Anchoring
Source: Biology (Basel). 2024 Dec 18;13(12):1062. doi: 10.3390/biology13121062 (PMC11673831; doi:10.3390/biology13121062)
Supplement: Supplementary file 1 [file biology-13-01062-s001.zip › File 1 - Guide to the supplemental material.pdf]

Guide to the supplemental material

The video file is a Metascan of a plaster cast of the footprint made when a latex artificial foot was used to "walk" over mud. On the plantar surface of this foot you can see a small ridge, this is the imprint of the wire leading to the attached strain gage.

The pdf file shows two raw screen shots from the data acquisition system. This data file is of a detached alligator foot wired with strain gages being "walked" over a force plate. The light blue traces are the data from the force plate. The second image is zoomed in on one of the walking episodes showing the response of the four different strain gages.

The EXCEL file contains the raw data record from the same file as the screen shots.
