# Supplementary material for: Forces Acting on the Foot of the American Alligator (Alligator mississippiensis) During Pedal Anchoring
Source: Biology (Basel). 2024 Dec 18;13(12):1062. doi: 10.3390/biology13121062 (PMC11673831; doi:10.3390/biology13121062)
Supplement: Supplementary file 1 [file biology-13-01062-s001.zip › File 5 - Supplemental material screen shots.pdf]

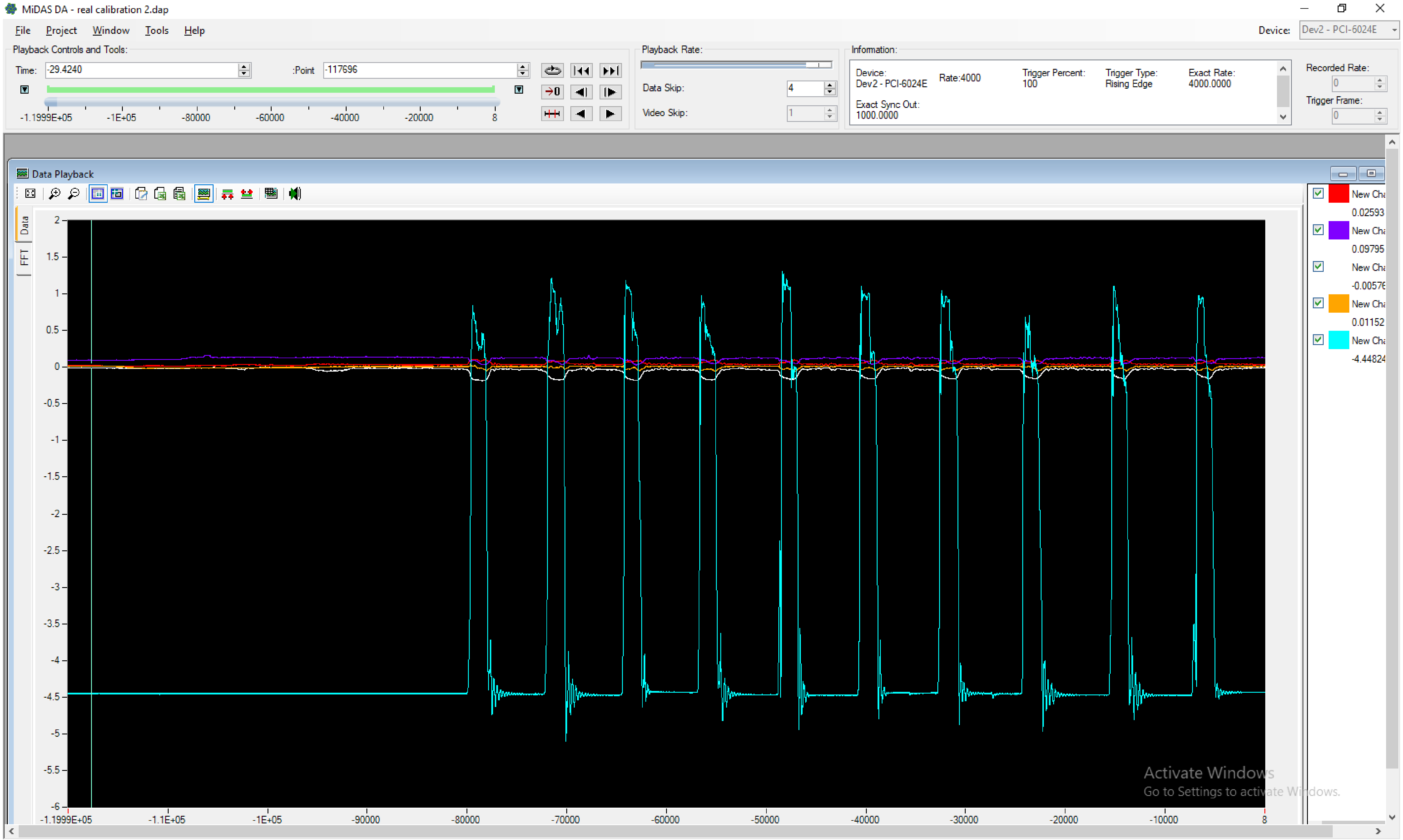
MiDAS DA - real calibration 2.dap
File
Project
Window
Tools
Help
Device:
Dev2 - PCI-6024E
Playback Controls and Tools:
Time:
-29.4240
:Point
-117696
-1.1999E+05
-1E+05
-80000
-60000
-40000
-20000
8
Playback Rate:
Data Skip:
4
Video Skip:
1
Information:
Device:
Dev2 - PCI-6024E
Rate:4000
Trigger Percent:
100
Trigger Type:
Rising Edge
Exact Rate:
4000.0000
Exact Sync Out:
1000.0000
Recorded Rate:
0
Trigger Frame:
0
Data Playback
Data
FFT
2
1.5
1
0.5
0
-0.5
-1
-1.5
-2
-2.5
-3
-3.5
-4
-4.5
-5
-5.5
-6
-1.1999E+05
-1.1E+05
-1E+05
-90000
-80000
-70000
-60000
-50000
-40000
-30000
-20000
-10000
8
New Cha
0.02593
New Cha
0.09795
New Cha
-0.00576
New Cha
0.01152
New Cha
-4.44824
Activate Windows
Go to Settings to activate Windows.

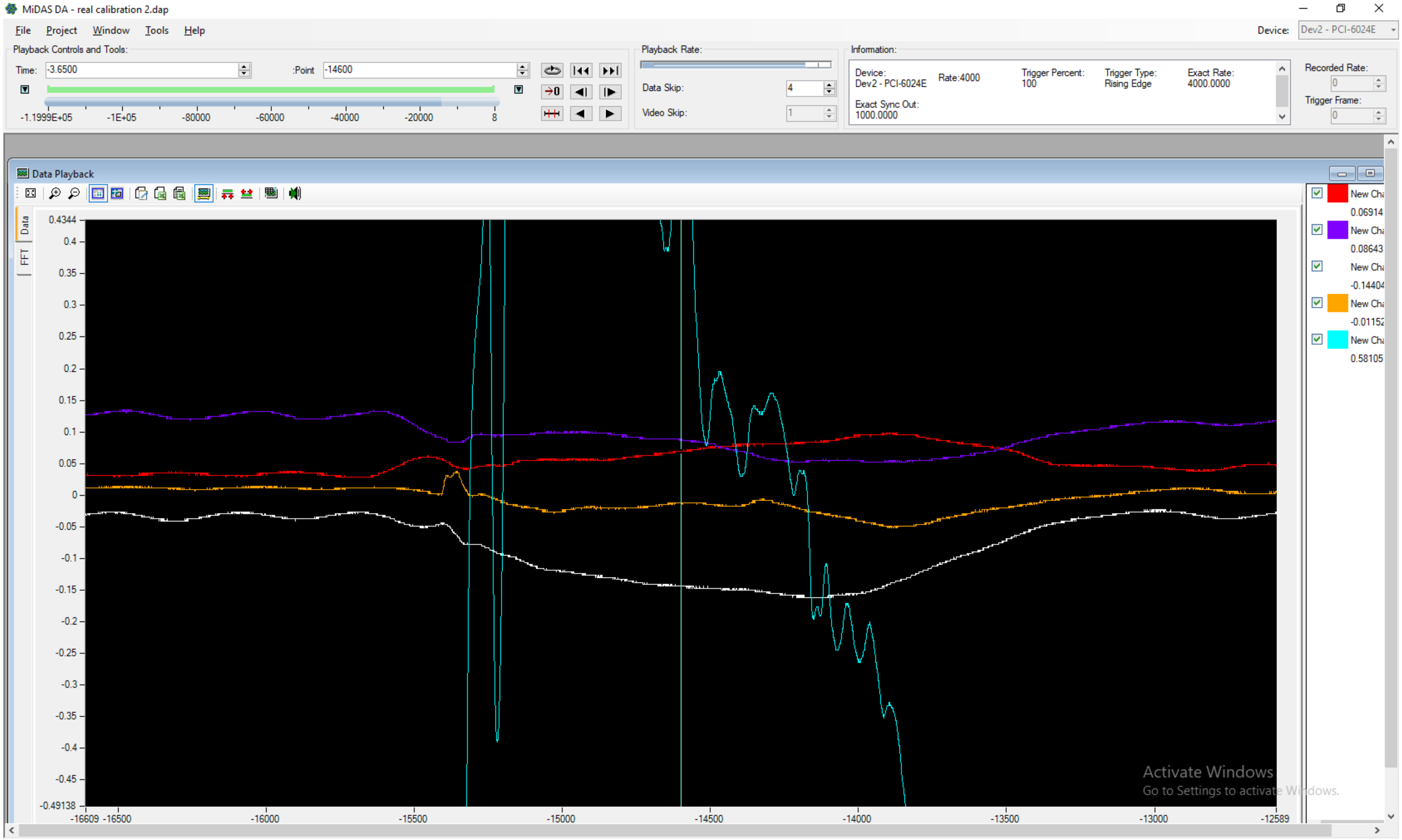

MiDAS DA - real calibration 2.dap
File
Project
Window
Tools
Help
Device:
Dev2 - PCI-6024E
Playback Controls and Tools:
Time:
-3.6500
:Point
-14600
-1.1999E+05
-1E+05
-80000
-60000
-40000
-20000
8
Playback Rate:
Data Skip:
4
Video Skip:
1
Information:
Device:
Dev2 - PCI-6024E
Rate:4000
Trigger Percent:
100
Trigger Type:
Rising Edge
Exact Rate:
4000.0000
Exact Sync Out:
1000.0000
Recorded Rate:
0
Trigger Frame:
0
Data Playback
Data
FFT
0.4344
0.4
0.35
0.3
0.25
0.2
0.15
0.1
0.05
0
-0.05
-0.1
-0.15
-0.2
-0.25
-0.3
-0.35
-0.4
-0.45
-0.49138
-16609
-16500
-16000
-15500
-15000
-14500
-14000
-13500
-13000
-12589
New Cha
0.06914
New Cha
0.08643
New Cha
-0.14404
New Cha
-0.01152
New Cha
0.58105
Activate Windows
Go to Settings to activate Windows.
